# Multiple Retinal Anomalies in Wfs1-Deficient Mice

**DOI:** 10.3390/diagnostics10090607

**Published:** 2020-08-19

**Authors:** Arleta Waszczykowska, Agnieszka Zmysłowska, Marcin Braun, Marilin Ivask, Sulev Koks, Piotr Jurowski, Wojciech Młynarski

**Affiliations:** 1 Department of Ophthalmology and Vision Rehabilitation, Medical University of Łódź, Żeromskiego 113 Street, 90-549 Łódź, Poland; arleta.waszczykowska@umed.lodz.pl (A.W.); piotr.jurowski@umed.lodz.pl (P.J.); 2 Department of Clinical Genetics, Medical University of Łódź, Pomorska 251 Street, 92-213 Łódź, Poland; agnieszka.zmyslowska@umed.lodz.pl; 3 Department of Pathology, Medical University of Łódź, Pomorska 251 Street, 92-213 Łódź, Poland; braunmarcin@gmail.com; 4 Department of Pathophysiology Institute of Biomedicine and Translational Medicine, University of Tartu, Ravila 19, 50411 Tartu, Estonia; marilin.ivask@ut.ee (M.I.); Sulev.Koks@ut.ee (S.K.); 5 Department of Pediatrics, Oncology and Hematology, Medical University of Łódź, Sporna 36/50 Street, 91-738 Łódź, Poland

**Keywords:** Wolfram syndrome, wolframin, *Wfs1*, Wfs1KO mouse model, proliferative retinopathy

## Abstract

Background: Wolfram syndrome (WFS, OMIM: #222300) is an ultrarare autosomal recessive disorder characterized by diabetes insipidus, diabetes mellitus, optic nerve atrophy and deafness. It has been reported that the average retinal thickness in WFS patients decreases with the progression of the disease. Aim: To investigate retinal thickness and wolframin expression disorders in Wolfram syndrome 1 gene knockout (Wfs1KO) mice compared to their wild-type (WT) littermates. Materials and methods: Both bulbs with optic nerves of three mice Wfs1WT and three Wfs1KO were taken for the histopathological examination. A strain of knockout mice with mutation in exon 8 was used. Results: No expression of wolframin protein in the retina and neurodegeneration of the optic nerve of Wfs1KO mice as compared among Wfs1WT mice was observed. The mean central retinal thickness was thinner and the retinal thickness/longitudinal diameter ratio was significantly lower in hte Wfs1KO as compared to the Wfs1WT mice. In four (67%) eyeballs of Wfs1KO mice, intra-retinal neovessels were observed. Conclusions: Wfs1KO mice retina with mutation in exon 8 present similar clinical features as patients with WFS in the form of reduced retinal thickness and neurodegeneration of the optic nerve. The presence of proliferative retinopathy observed in Wfs1KO mice requires further investigation.

## 1. Introduction

Wolfram syndrome (WFS, OMIM: #222300) is an ultrarare autosomal recessive disorder characterized by diabetes insipidus (DI), diabetes mellitus (DM), optic nerve atrophy (OA), and deafness (D)–DIDMOAD. WFS is caused by mutations in the *WFS1* gene, which consists in 8 exons and is located in the fourth chromosome 4p16.1 region [1]. 

We have previously described that the average retinal thickness in WFS patients decreases with the progression of the disease [2]. 

To date, mutant mice lacking the *Wfs1* gene have been generated in three independent laboratories [3,4,5]. One strain of mice is a conditional mutant with the specific targeting of the *Wfs1* gene in pancreatic islet beta cells [5]. The other two strains were generated by inserting a neomycin-resistant cassette into the gene coding area. They only differed in the exact target region and background of the strain. Ishihara et al. traced the exon 2 gene, Kõks et al. exon 8 [2,3,4]. 

In the present study, for the first time we wanted to confirm our observations in a mouse model of Wolfram syndrome. The *Wfs1*-deficient mice used in the current study had the exon 8 disrupted resulting in the deletion of amino acids 360–890. In the literature, this strain is suggested as showing the most similar clinical features as patients with WFS [6]. 

We investigated the retinal thickness and wolframin expression disorders in Wolfram syndrome 1 gene knockout (Wfs1KO) mice compared to their wild-type (WT) littermates. 

## 2. Material and Methods

The idea to perform our study came from the fact that during a routine annual examination of our nationwide cohort of WFS patients, one patient was found to have with corneal abnormalities. Thus, we conducted a corneal evaluation study in all our patients with Wolfram syndrome and in most of them we found corneal changes similar to keratoconus. In order to confirm our observations and the fact that corneal changes in this case might depend on the gene and not the species, we carried out experimental tests on a mouse model [7]. Similarly, in our previous studies, we have documented that progressive retinal thinning is present among patients with Wolfram syndrome [2]. Thus, the number of mice we evaluated in the current study was low because the design of our project has only a confirmatory not an exploratory approach.

### 2.1. Animals

Wfs1KO mice were generated by invalidating the eighth exon of the *Wfs1* gene as described previously [4]. Experiments were performed with Wfs1KO male mice with a 129S6/SvEvTac background and their wild-type (Wfs1WT) littermates. The mice were kept in groups of eight per cage at 22 ± 1 °C in a room illuminated artificially from 7 a.m. to 7 p.m. Tap water and food pellets were freely available. 

In our approach, we decided to match the age of the mice to the median range of age of our study human cohorts (17.2–20.4 years [7,8,9]) according to two formulas which consider the average lifespan of laboratory mice and puberty [10]. The median age of mice of 109 days corresponds to the human age from 11.9 years (based on lifespan formula) to 29.9 years (based on puberty age formula). If we merged these two formulas, the age of our mice corresponds to 18.8 years in men, which mostly resembled the median age observed in our cohort of patients with Wolfram syndrome. Based on this calculation, we decided to scarify mice between 107 and 111 days of life to perform our study. Three mice Wfs1WT and three Wfs1KO were sacrificed at the age of 109 ± 2 days and both bulbs of the eye along with optic nerves were taken for the histopathological examination. 

Immunohistochemical reagents for the preparation of histopathological specimens were purchased from Leica Biosystems (Wetzlar, Germany) and from Sigma-Aldrich (St. Louis, MO, USA).

Permission for this study was given by the Estonian National Board of Animal Experiments (No. 86, 4 May 2016) in accordance with the European Communities Directive of September 2010 (2010/63/EU).

### 2.2. Histopathological Examination

The bulbs of the eyes, after fixation in 10% neutral buffered formalin for at least 24 h and after macroscopic evaluation, were processed into tissue blocks embedded in paraffin (FFPE). Six consecutive 4 μm-thick sections were taken at the largest longitudinal diameter of each bulb and then stained with hematoxylin and eosin. The most representative sections were selected by standard light microscopy (Light Microscope BX43, OLYMPUS Europa SE & CO, Hamburg, Germany). The morphological analysis of the retina was performed using the UltraFast Scanner (Philips IntelliSite Solution, Best, The Netherlands) with DigiPath™ Professional Production Software (Xerox, Norwalk, CT, USA).

In the morphological examination, the following qualitative and quantitative parameters were selected for the examination: longitudinal diameter of the eye (um) (optic nerve-to-cornea), lateral diameter of the eye (um) (cornea–cornea), retinal thickness as mean of three consecutive measurements around the optic nerve (um), retinal thickness/eye longitudinal diameter ratio, retinal lateral thickness/eye longitudinal diameter ratio, retinal inflammation presence (YES/NO), retinal fibroblast reaction presence (YES/NO), signs of retinal degeneration (YES/NO), retinal neovascularization (YES/NO) [11,12,13,14,15,16]. 

### 2.3. Immunohistochemistry

The immunohistochemical expression of WFS1 (Wolfram Syndrome Protein 1), GFAP (Glial Fibrillary Acidic Protein) and NSE (Neuron Specific Enolase) was evaluated following the standard protocol [17,18]. To perform specific staining, the eyeballs were cut into 5 μm thick cross-sections (Accu-Cut SMR 200 rotary microtome, Sakura Finetek, Tokyo, Japan) and collected on polylysine-covered microscopic slides. The specimens were tested for antibody presence: for WFS1 (Catalog number: 11558-1-AP, Immunogen Catalog Number: AG2114, WFS1 Polyclonal Rabbit Antibody, Proteintech Group Inc, Rosemont, IL, USA), for GFAP (M0761, Clone GF2, Monoclonal Mouse Anti-Human with cross-reactivity to mice, Dako-Agilent, San Jose, CA, USA), for NSE (Clone BBS/NC/VI-H14, Monoclonal Mouse Anti-Human with cross-reactivity to mice, Dako-Agilent, San Jose, CA, USA). GFAP and NSE antigens were unmasked by incubating the sections in pH 8.0 citrate buffer (S1699, Dako-Agilent, San Jose, CA, USA) in PT-link (Dako-Agilent, San Jose, CA, USA) for 20 min at 97 °C. The WFS1 antigen was unmasked by incubating the sections in pH 6.0 citrate buffer (S1699, Dako-Agilent, San Jose, CA, USA) in a water bath for 30 min at 98 °C. The visualization of the result was done using specific detection systems—EnVisionTM FLEX+ (Dako-Agilent, San Jose, CA, USA). The reactions were carried out using the Autostainer Link (Dako-Agilent, San Jose, CA, USA). For the detection of the antibody, EnVision+ System-HRP with DAB as a chromogen (K4011, Dako-Agilent, San Jose, CA, USA) were used according to the instructions of the manufacturer. Nuclei were counterstained with hematoxylin.

The stained sections were evaluated qualitatively under light microscopy (Light Microscope BX43, OLYMPUS Europa SE & CO, Hamburg, Germany). Representative images were taken using UltraFast Scanner (Philips IntelliSite Solution, Eindhoven, The Netherlands) with DigiPath™ Professional Production Software (Xerox, Norwalk, CT, USA).

### 2.4. Statistical Analysis

For the statistical calculations of the histopathological examination results, each eye from each analyzed group was treated separately, but without a division into right and left eyes. Therefore, six measurements were obtained in both the study and control groups. 

Continuous variables were presented as medians followed by interquartile ranges (IQR), while nominal variables were presented as numbers followed by percentages in brackets. The Shapiro–Wilk test was used to assess the normality of distribution. Continuous variables were compared using the Mann–Whitney U-test in the case of a non-normal distribution or t-test in the case of normal distribution. The Statistica 12.5 PL package (Statsoft, Tulsa, OK, USA) was used for the analysis. *p* values < 0.05 were considered statistically significant.

## 3. Results

### 3.1. Morphometric Analysis of Mice Retinal Thickness and Morphology

The mean longitudinal diameter of the eye and the lateral diameter of the eye in the Wfs1KO group were 2921 ± 125 μm and 2616 ± 172 μm, respectively. The corresponding values in the Wfs1WT mice group were 2868 ± 63 μm and 2608 ± 111 μm, respectively. These values did not differ between groups. 

**Figure 1:**
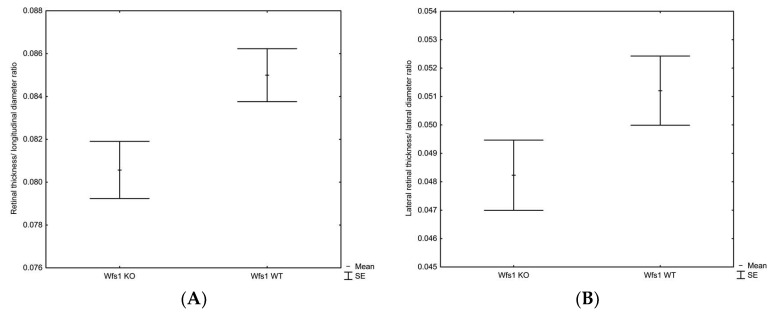
Box-and-whiskers plot of the mean retinal thickness/longitudinal diameter ratio (**A**) and the lateral retinal thickness/lateral diameter ratio (**B**) in the Wfs1KO and Wfs1WT mice, Mann–Whitney *U* test. Abbreviations: Wfs1KO: Wolfram syndrome 1 gene knockout mice; Wfs1WT: Wolfram syndrome 1 gene wild-type mice.

The mean central retinal thickness was slightly thinner in Wfs1KO as compared to the Wfs1WT mice (235 ± 8 μm vs. 244 ± 5 μm, *p* = 0.058, respectively). The retinal thickness/longitudinal diameter ratio in the Wfs1KO mice was significantly lower compared to the Wfs1WT mice (0.081 ± 0.003 vs. 0.085 ± 0.003, *p* = 0.035, respectively), whereas the lateral retinal thickness/lateral diameter ratio was only marginally lower 0.048 ± 0.003 vs. 0.051 ± 0.003, *p* = 0.11, respectively). These results are illustrated in Figure 1A,B.

**Figure 2:**
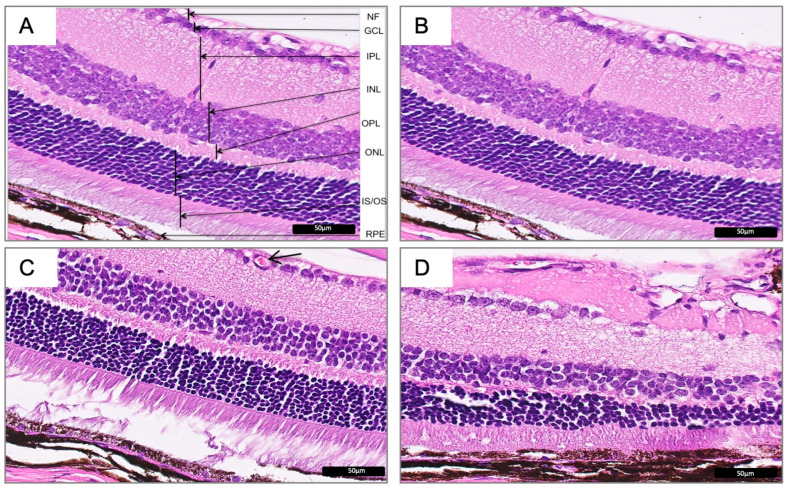
Hematoxylin and eosin images representing normal mice retina in Wfs1-wild-type mice (**A**,**B**) and the corresponding representative images of the defect mice retina in the Wfs1-mutated mice (**C**,**D**). The neovascularization (**C**, **arrow**) and morphological (**D**) defects can be seen. Magnification of 400X. Abbreviations: Wfs1: Wolfram syndrome 1 gene mice; NF: nerve fiber layer; GCL: ganglion cell layer; IPL: inner plexiform layer; INL: inner nuclear layer; OPL: outer plexiform layer; ONL: outer nuclear layer-representing photoreceptor cell bodies; IS/OS: inner segment/outer segment; RPE: retinal pigment epithelium.

The comparative analysis of Wfs1KO and Wfs1WT mouse retina (H&E) images revealed significant histomorphological differences in all the retinal layers between samples (Figure 2). 

In all Wfs1KO cases, the inner and outer retina did show significant abnormalities. There were observable loosely arranged cells junctions, a disordered arrangement of cells in each layer and a reduced thickness of segments.

In four (67%) eyeballs of Wfs1KO mice, the intra-retinal neovessels in ganglion cell layer (GCL) was observed. The vessel dilation or microaneurysms were found in three animals. None of these findings were seen among the Wfs1WT mice (Figure 2C).

No symptoms of retinal inflammation and retinal fibroblast reaction in any of the analyzed subgroups were observed. Seven eyeballs showed symptoms of retinal degeneration (two eyeballs of Wfs1WT mice and five eyeballs of Wfs1KO mice).

### 3.2. Immunohistochemistry Analysis

**Figure 3:**
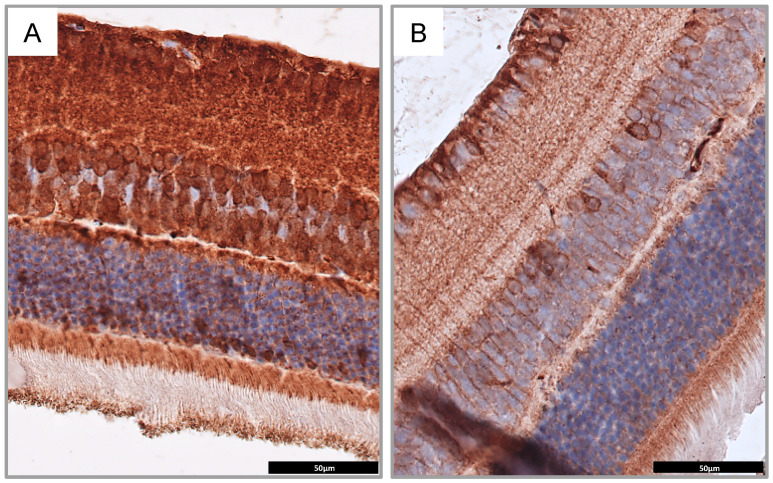
Immunohistochemical staining for wolframin in Wfs1-wild-type mice (**A**) and Wfs1-mutated mice (**B**) showing significantly less intensive staining in the latter. Magnification of 400×. Abbreviations: *Wfs1*, Wolfram syndrome 1 gene mice.

**Figure 4:**
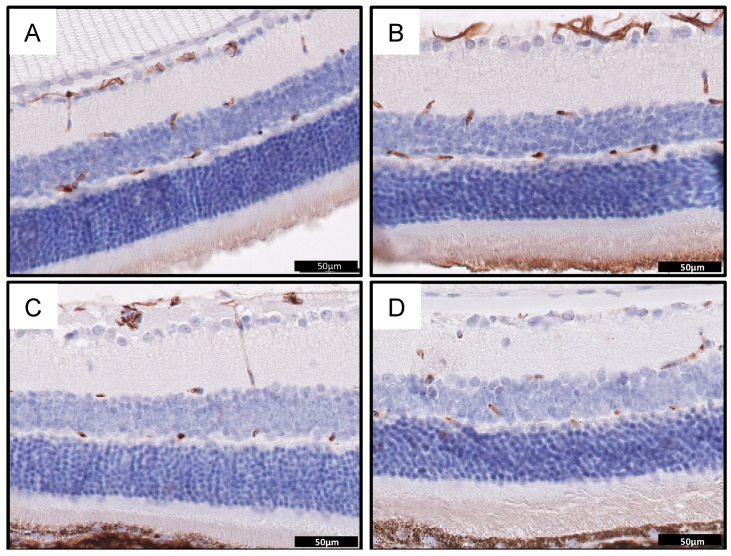
Immunohistochemical staining for glial fibrillary acidic protein (GFAP) showing retinal neuronal network in normal mice retina in Wfs1-wild-type mice (**A**,**B**) and the corresponding representative images of neuronal retinal defects in the Wfs1-mutated mice (**C**,**D**). Fewer neurons positive for GFAP can be seen in (**C**,**D**). Magnification of 400X. Abbreviations: GFAP: glial fibrillary acidic protein; *Wfs1*: Wolfram syndrome 1 gene mice.

In this study, performed on the mouse model of the Wolfram syndrome, we confirmed that the wolframin protein is not expressed in retina of Wfs1KO mice when compared to mice with the normal *Wfs1* gene (Wfs1WT) (Figure 3A,B). Moreover, the staining for GFAP as a neuronal marker showed nerve degeneration in Wfs1KO mice, confirming that this mouse model presented similar ophthalmological features to these observed among patients with WFS (Figure 4). 

The Wfs1KO mice had significantly fewer GFAP-positive cells than the Wfs1WT animals. All the GFAP-positive cells in the Wfs1KO group had a lower staining intensity than the controls. The distribution of the GFAP immunoreactivity in both analyzed groups of mice was located in the same retinal layers: in the nerve fiber (NF), ganglion cell (GCL), inner plexiform (IPL), inner nuclear (INL), outer plexiform (OPL), and outer nuclear (ONL) layers—representing the ganglion cell nerve fibers, ganglion cells, amacrine cells, horizontal cells, photoreceptor cell bodies layers as well as the Müller glial cells, astrocytes and microglia. However, a significantly lower number of GFAP positive cells in the inner nuclear layer in Wfs1KO retinas was observed (Table 1, Figure 4).

## 4. Discussion

Previous studies on the ocular wolframin expression were focused in healthy human and animal models. The presence of wolframin in the retinas including retinal pigment epithelium, retinal ganglion cells, optic axons and the proximal optic nerve has been proven. 

To the best our knowledge, there is only one study investigating the wolframin expression in the Wfs1KO mice retinas in the literature. The described study was carried out on mice in which the exon 2 was modified [19]. 

Human studies have revealed that the *WFS1* gene mutations are mostly located in exon 8 and the sequence first exon 8 for molecular diagnosis seems to be the most appropriate [20]. In this study we used mice with the exon 8 modified in the age of 3 months. At this age, Wfs1KO animals already display a characteristic phenotype for the WFS [21].

The results of our experimental studies on the Wolfram syndrome mouse model confirm our previous like optical coherence tomographic (OCT) results in WFS patients. We have previously described that the average retinal thickness decreases with the progression of the disease. An important observation was that the average retinal thickness and total retinal volume of the OCT parameters in the WFS patients have been decreasing slower than the retinal nerve fiber layer (RNFL) value [2]. Analyzing the results of the present study on Wfs1KO mice we can explain this phenomenon by the fact that intercellular connections are loosened in all the nerve layers of the retina and therefore, despite significant nerve cell degeneration, the average retina thickness may decrease more slowly. As in humans, we saw a significant reduction of retinal thickness/longitudinal diameter ratio in the Wfs1KO mice compared to the WT littermates. 

A surprising observation for us was finding retinal neovascularization in 67% of the Wfs1KO mouse retinas. According to our knowledge, only three studies report the occurrence of diabetic retinopathy in WFS patients [22,23,24]. 

Present molecular knowledge of the WFS allows for the statement that wolframin—as a product of the *WFS1* gene—is an integral component of the endoplasmic reticulum (ER). The loss of *WFS1* leads to neurodegeneration through apoptosis due to increased ER stress, which is generally considered to induce oxidative stress [3,25]. It is well known that inflammation, oxidative stress, and neuronal dysfunction are also contributing to the pathogenesis of ischemic retinal diseases [26]. The most widely used model to study retinal ischemic diseases is the mouse oxygen-induced retinopathy (OIR) model [27]. In addition to angiogenesis disorders in OIR, neuronal damage has also been observed and pathological effects on the retinal glia have also been reported [28]. The interaction between pathologic retinal angiogenesis and retinal glial dysfunction plays a key role in the progression of OIR [29]. Retinal astrocytes, which are located in the layer of nerve fibers, are critical for the proper development of retinal vessels. Since retinal astrocytes are damaged in the retina of the OIR model, their VEGF-A secretion causes pathological neovascularization [30]. In the retina of the eye of the mouse, in response to the injury caused by ischemia, the amount of microglia increases [31] and plays an important role in organizing blood vessel formation [32].

Our results are concordant with the previous studies of the OIR mouse model because we also observed that microglial cells in the GFAP staining were attached to the neovascular tufts. Therefore, among the causes of observed retinal neovascularization in our Wfs1KO mice, should be considered the consequences of chronic ER stress and neurodegeneration, but we cannot exclude hyperglycemia, as in classical diabetic retinopathy. Unfortunately, in our study we did not perform a glycemic test.

Our study has some limitations. Most importantly, it was performed on a small number of Wfs1KO mice eyes. In addition, the lack of long-term studies of Wfs1KO mice at different ages and thus at different stages of the disease prevented us from observing the time of retinal thickness changes appearance. Our detailed histological analyses using the quantitative morphometry of the mouse retina could be enriched by in vivo functional tests like optical coherence tomographic (OCT) imaging, fluorescein angiography (FA) and electroretinographic (ERG) examinations.

## Figures and Tables

**Table 1:** Morphometric analysis of the mice retinal thickness and morphology in the Wfs1KO and Wfs1WT mice. Abbreviations: Wfs1KO: Wolfram syndrome 1 gene knockout mice; Wfs1WT: Wolfram syndrome 1 gene wild-type mice; GFAP: glial fibrillary acidic protein.

Characteristics	Wfs1KO Male Mice	Wfs1WT Male Mice	*p*-Level *
Analyzed eyeballs/animals	*N* = 6/*N* = 3	*N* = 6/*N* = 3	NA
Longitudinal diameter of eye (μm)	2921 ± 125	2868 ± 63	0.376
Lateral diameter of eye (μm)	2616 ± 172	2608 ± 111	0.927
Retinal thickness (μm)	235.2 ± 8.0	243.7 ± 5.5	0.058
Retinal thickness/longitudinal diameter ratio	0.081 ± 0.003	0.085 ± 0.003	0.035
Lateral retinal thickness (μm)	125.8 ± 4.1	133.7 ± 11.5	0.135
Lateral retinal thickness/lateral diameter ratio	0.048 ± 0.003	0.051 ± 0.003	0.112
Intra-retinal neovessels in ganglion cell layer	4/3	0/6	0.110
Vessel dilation or microaneurysms	4/3	0/6	0.11
GFAP positive cells in inner nuclear layer (cells/100 μm^2^)	2.9 ± 0.3	3.8 ± 0.4	0.042

* significant difference according to the nonparametric tests. NA, not applicable.
